# Fabrication of High-κ Dielectric Metal Oxide Films on Topographically Patterned Substrates: Polymer Brush-Mediated Depositions

**DOI:** 10.1021/acsami.2c07966

**Published:** 2022-07-07

**Authors:** Pravind Yadav, Riley Gatensby, Nadezda Prochukhan, Sibu C. Padmanabhan, Arantxa Davó-Quiñonero, Philip Darragh, Ramsankar Senthamaraikannan, Bríd Murphy, Matthew Snelgrove, Caitlin McFeely, Sajan Singh, Jim Conway, Robert O’Connor, Enda McGlynn, Ross Lundy, Michael A. Morris

**Affiliations:** † AMBER Research Centre and School of Chemistry, Trinity College Dublin, Dublin 2, Ireland; ‡ School of Physical Sciences, Dublin City University, Glasnevin, Dublin 9, Ireland; § National Centre for Plasma Science and Technology, Dublin City University, Glasnevin, Dublin 9, Ireland

**Keywords:** ultrathin films, high-κ dielectric, polymer brush, conformal deposition, ion infiltration

## Abstract

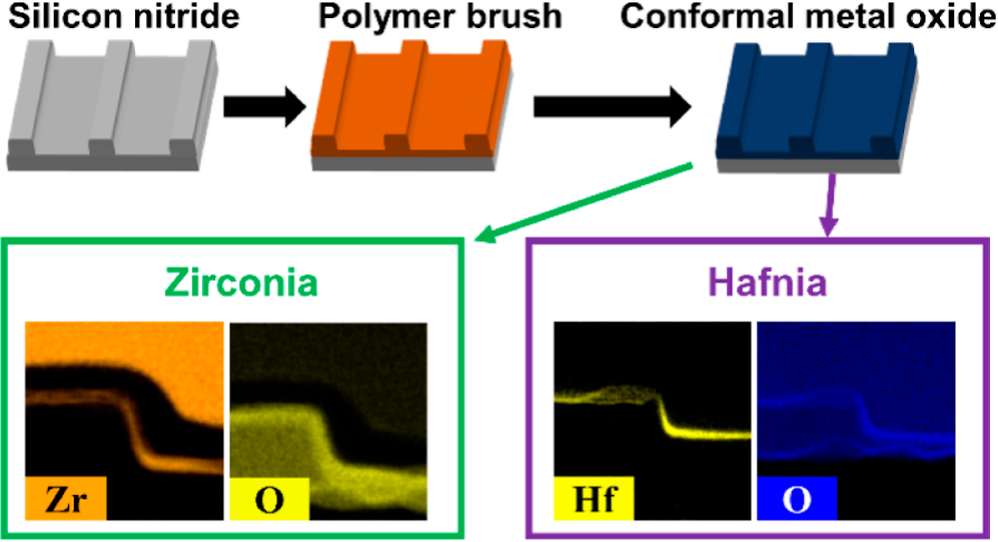

Fabrication of ultrathin films of dielectric (with particular reference to materials with high dielectric constants) materials has significance in many advanced technological applications including hard protective coatings, sensors, and next-generation logic devices. Current state-of-the-art in microelectronics for fabricating these thin films is a combination of atomic layer deposition and photolithography. As feature size decreases and aspect ratios increase, conformality of the films becomes paramount. Here, we show a polymer brush template-assisted deposition of highly conformal, ultrathin (sub 5 nm) high-κ dielectric metal oxide films (hafnium oxide and zirconium oxide) on topographically patterned silicon nitride substrates. This technique, using hydroxyl terminated poly-4-vinyl pyridine (P4VP-OH) as the polymer brush, allows for conformal deposition with uniform thickness along the trenches and sidewalls of the substrate. Metal salts are infiltrated into the grafted monolayer polymer brush films via solution deposition. Tailoring specific polymer interfacial chemistries for ion infiltration combined with subsequent oxygen plasma treatment enabled the fabrication of high-quality sub 5 nm metal oxide films.

## Introduction

Miniaturization of on-chip electronic components has driven the development of the semiconductor industry for the past half century. These advances have been enabled through top-down approaches by the use of deep UV (DUV) (193 nm) immersion technology combined with multiple patterning techniques such as litho-etch–litho-etch.^1−3^ Continued miniaturization below the 7 nm node has however resulted in cost and integration difficulties.^4,5^ Area selective deposition (ASD) techniques afford the opportunity to complement conventional photolithography.^6,7^ ASD can allow the fabrication of nanoscale material patterns at significantly lower cost and most importantly shorter processing times, compared to top-down lithography techniques.^8,9^

ASD is a patterning technique with excellent potential to complement existing optical lithography (DUV and EUV).^6,10^ ASD facilitates material deposition in a very controlled manner at pre-defined regions of a substrate by masking or area (de)activation. The ability of the ASD approach to deposit highly ordered metal and oxide films is advantageous because it can provide routes to produce material patterns that complement silicon device processing technology, especially in monolithic integration.^11^ Several cutting edge ASD technologies have been developed using chemical modification of the substrate surface with self-assembled monolayers (SAMs)^6,12^ and unreactive polymers to include the material on the region of interest using atomic layer deposition (ALD)^10,12−17^ or molecular layer depositions.^12,18,19^ For example, Bent et al. have proven the capacity of SAM to selectively deposit materials by activating and deactivating copper–silicon line space patterns.^6,7^ Furthermore, they demonstrated ALD deposition of Pt onto three-dimensional nanostructures using ion implantation of fluorocarbons (CF_*x*_).^20^ Similarly, Leskelä et al. devised a straightforward method for selectively depositing SAM on copper substrates in order to carry out material infiltration on silicon.^10^ Parsons et al. demonstrated the ALD approach for coating materials on specific substrate locations by using amorphous carbon patterning^21^ and exploiting intrinsic substrate selectivity.^22^

Our previous work described the use of a polymer brush film to selectively pattern material onto Cu/SiO_2_ line space patterns for ASD of Cu metal layers.^23,24^ We developed simple methods for the rapid grafting of end-terminated polymer brush films with complete coverage and subsequent conversion to numerous oxides (Al_2_O_3_, Co_3_O_4_) via liquid phase metal salt infiltration.^24,25^ Furthermore, we studied the precise parameters that control the monolayer formation of the polymer brush film. Polymer brush layer deposition requires accurate optimization of polymer molecular weight, casting solution concentration, and reactive terminal group density in the formation of pinhole free high coverage monolayers. A vapor phase volatile organometallic titanium(IV) isopropoxide precursor infiltration of monolayer brush film was studied using a simple apparatus.^26^ In addition to this, a surface deactivation strategy using an unreactive hydroxyl terminated polystyrene (PS-OH) brush to prevent metal ion inclusion was demonstrated.^23^

A significant advantage of the polymer brush technique over SAM depositions is the fast processing speed (a few minutes for polymer brush grafting vs hours to days for SAM’s monolayer coating). Furthermore, the polymer brush-assisted deposition process overcomes the issues that the ALD technique poses such as the line of sight deposition and the possibility of uniformly coating trench corners or sidewalls.^27^ So far, the polymer brush technique has been shown to facilitate the deposition of metal oxide films uniformly across relatively smooth substrate surfaces. The application of this technique throughout the trenches and pitches of a topographically patterned substrate would dramatically widen the use case scenarios and allow additional device integration and provide advantages over current methodologies. Using polymer brush aided conformal deposition of high-κ materials across trenches would be beneficial for developing 3D fin field-effect transistors (FinFETs).^28^ FinFETs are multigate devices in which the gate wraps the device channel. High-κ dielectric materials such as HfO_2_ and ZrO_2_ offer low gate leakage current and prevent power dissipation, making them the materials of choice in advanced complementary metal oxide semiconductor technology.^29^ Furthermore, HfO_2_ and ZrO_2_ are the potential ferroelectric materials that could be effective in the fabrication of sub 7 nm FinFET device architecture.^30^

In this work, we demonstrate a straightforward, robust approach for liquid phase infiltration of zirconium oxynitrate and hafnium chloride onto the silicon nitride trenches using P4VP-OH grafted brushes. In order to deposit thin metal oxide films, it is critical to form a high coverage polymer brush template such as hydroxyl terminated P4VP to facilitate inorganic precursor uptake. Furthermore, other reactive polymers such as P2VP and polymethyl methacrylate (PMMA) were infiltrated with the metal precursors to compare the results. Applicability of the reactive polymer system and favorable interactions with the metal cations on native oxide silicon substrates have been discussed in the literature.^31,32^ This study demonstrates the polymer deposition across the trenches for realizing conformal coating of ZrO_2_ and HfO_2_. In addition to this, a surface deactivation strategy using an unreactive PS-OH brush to prevent metal ion inclusion is shown.^33,34^

This research work prototypes the deposition of HfO_2_ and ZrO_2_ thin films at moderate temperatures (200–250 °C) which is significant due to the low process temperature required (<400 °C) for front and back end of line fabrication.^35^ The fundamental insights showcase the ways to implement polymer brush lithography for the fabrication of high-κ metal oxides such as HfO_2_ and ZrO_2_. This method could be further used in the fabrication of next generation logic gate devices or ferroelectric devices such as ferroelectric random-access memory and FETs.

## Experimental Section

### Materials

Silicon nitride-coated substrates were prepared using a low-pressure chemical vapor deposition (CVD) on p-type silicon substrate with ∼7 nm thick SiO_2_ layer. The topographically patterned Si_3_N_4_ coated substrate was fabricated using 193 nm photolithography and processed with photoresist and dry etch technique with pitches ranging from 75 nm to 1 μm and a trench depth of 40 nm.

#### Functionalized Polymers

Hydroxyl terminated poly-4-vinyl pyridine (P4VP-OH) 5 kg mol^–1^ (PDI: 1.28), poly-2-vinyl pyridine (P2VP-OH) 6.2 kg mol^–1^ (PDI: 1.05), PMMA-OH 6.3 kg mol^–1^ (PDI: 1.03), and polystyrene (PS-OH) 6 kg mol^–1^ (PDI: 1.05) were purchased from Polymer Source (Canada) and used without further purification.

#### Solvents

High-performance liquid chromatography grade acetone, tetrahydrofuran (THF), ethanol, isopropyl alcohol (IPA), and toluene were purchased from MERCK Ireland and used without further purification.

#### Metal Precursors

Hafnium(IV) chloride and zirconium(IV) oxynitrate hydrate were purchased from Sigma-Aldrich and used as received.

### Fabrication

**Figure 1:**
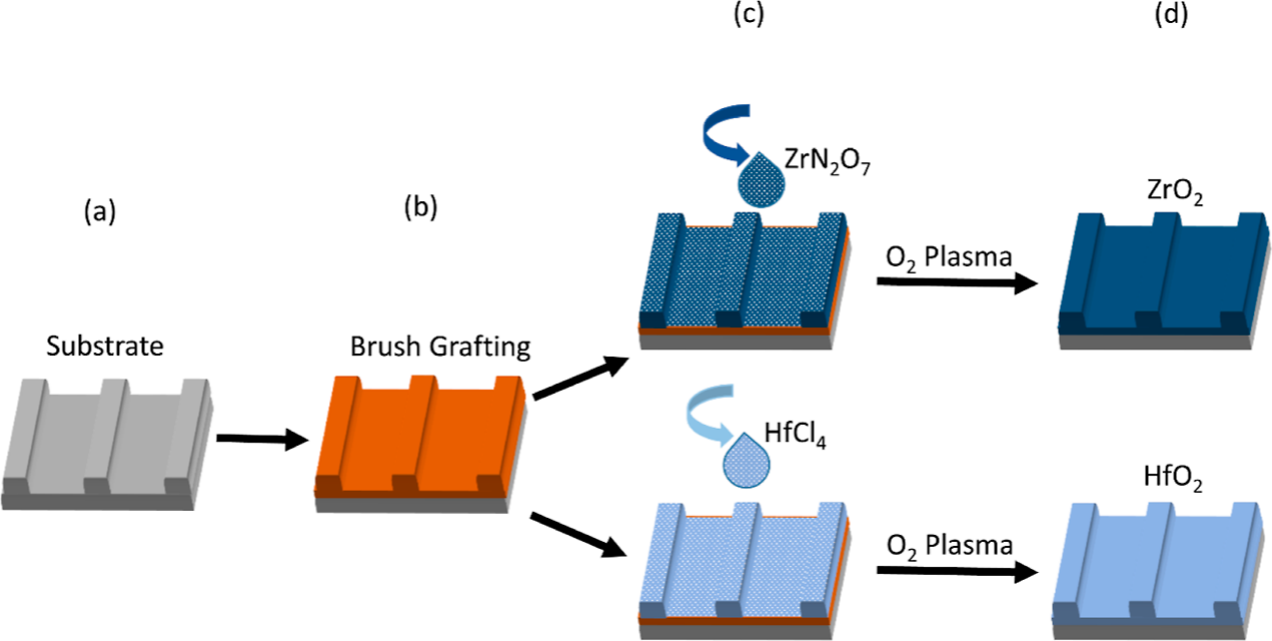
Schematic process flow of the sub-5 nm metal oxide film fabrication process. (a,b) Monolayer polymer brush film grafting on topographically patterned substrate, (c) inorganic ion infiltration, and (d) oxidation of inorganic precursor and elimination of polymer.

Topographically patterned silicon nitride substrates were cleaved into 2 by 2 cm^2^ pieces and ultrasonically washed in IPA for 20 min. Silicon p-type ⟨100⟩ native oxide wafers were also used as substrates to optimize polymer brush grafting. Figure 1a indicates the degreasing and hydroxyl functionalization of the substrate surface using oxygen plasma treatments for 2 min (40 kHz, 50 W, Diener PICO Barrel Asher). Polymers were dissolved in their respective solvent [PS-OH in toluene, PMMA-OH in toluene, P2VP in THF, and P4VP in THF/IPA (3:2) mixture] based on Hansen solubility parameters^36,37^ and stirred overnight at 500 rpm to obtain a homogeneous 0.2 wt % solutions, which were then spin deposited onto the substrates at 3200 rpm for 30 s. Thereafter, samples were placed on a hotplate at 230 °C for 2 min to form covalent bonding between the end hydroxyl group of the polymer and the complementary functional group present on the substrate via condensation reactions.^25,26^ The samples were then ultra-sonicated in the relevant solvent to remove any physisorbed polymer and yield chemically grafted monolayer polymer films (see Figure 1b). A monolayer polymer film was then infiltrated with 0.5 wt % ethanolic solution of either hafnium(IV) chloride or zirconium(IV) oxynitrate (see Figure 1c) and subsequently underwent oxygen plasma treatment of 1 × 10^–2^ mbar of oxygen at 30 W for 20 min with an oxygen flow of 100 sccm to oxidize the metal precursor and eliminate the polymer brush layer (see Figure 1d), thus forming high-quality metal oxide films.

### P4VP Pellet Fabrications

P4VP homopolymer (unfunctionalized) powder was used to prepare reference substrates. The homopolymer was dry-pressed into disc-shaped pellets (≈2 mm thick) at 350 MPa in a 13 mm diameter steel pellet die (Specac, 13 mm evacuable pellet die). These pellet substrates were used to determine the baseline contact angles (CAs) for pure polymer surfaces to calculate brush coverage on Si substrates. Plasma cleaned SiO_2_ was used as a reference (60 s, 40 kHz, 50 W, Barrel Asher).

### Characterization

Angled (90°) scanning electron microscopy (SEM) images were obtained by field emission SEM (Zeiss Ultra) using secondary electron detector (in lens) with 1–5 kV accelerating voltage. Focused ion beam etching (FIB Helios nano lab 460) utilizing a capping layer of e-beam Pt (100 nm) and ion beam Pt (2 μm) was used to prepare lamellae using standard high kV milling and low kV polish which rendered the lamellae electron transparent indicating an appropriate thickness for transmission electron microscopy (TEM). TEM (FEI Orisis) sample analysis was carried out using bright field and scanning TEM (STEM) imaging. The detector lengths used in STEM were 220, 550, and 770 mm. The accelerating voltage was set at 200 kV. The energy-dispersive X-ray spectroscopy (EDX) beam current was 1 nA with an acquisition time of 30 min. Atomic force microscopy (AFM Park System XE7) was used with a noncontact cantilever (AC160TS, force constant ∼ 26 N·m^–1^) with resonant frequency ∼300 kHz.

Under ultrahigh vacuum conditions, X-ray photoelectron spectroscopy (XPS) data were collected using a Thermo Fisher-VG equipment equipped with an Al Kα (*h*ν = 1486.7 eV) X-ray source and a 3 channeltron hemispherical electron analyzer (base pressure; 1 × 10^–9^ mbar). Casa XPS program was used to analyze XPS data. The C 1s peak at 284.8 eV of adventitious carbon was used as a reference for the binding energy scale.^38^ An analyzer pass energy of 200 eV was used for survey scans, while a pass energy of 20 eV was set to recordhigh-resolution spectra of characteristic core levels. To ensure that the sample did not break vacuum after the polymer ashing process, the oxygen plasma process was performed in a purpose-built chamber with a custom-made plasma source coupled with the XPS analysis chamber. The plasma process took place in a pressure of 1 × 10^–2^ mbar of oxygen at 30 W for 20 min with an oxygen flow of 100 sccm. Dynamic CA measurements were taken at three representative regions of each sample using a high speed camera. A 60 Hz sampling rate was used to capture advancing and receding water CAs. Flow rates of 5 nL s^–1^ were used to dispense liquids through a 35 gauge needle (135 μm OD) with droplet volume ranging from 40 to 80 nL. Polymer brush coverage was determined using the Cassie Baxter equation (eq 1) from the water CA.

Thermogravimetric analysis (TGA) was performed using a PerkinElmer TGA on the homopolymer powder (P4VP-OH) in the temperature range of 30–600 °C for 60 min (see Figure S1).

## Results and Discussion

**Figure 2:**
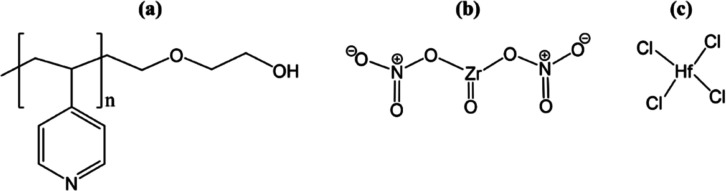
(a) Hydroxyl end terminated poly-4-vinyl pyridine, (b) zirconium oxynitrate, and (c) hafnium tetrachloride.

To prepare a uniform thin film of metal oxides (HfO_2_ and ZrO_2_), a polymer template approach was developed that facilitates controlled inclusion of the metal ions. We chose a P4VP-OH brush as the template for the metal oxide deposition as this polymer has high coordination toward metal ions.^39^ The Hansen approach was used to find the optimum solvent combination to solubilize the P4VP-OH polymer.^37^ The P4VP-OH (0.2 wt %) dissolved in a 60:40 THF/IPA mixture is then spin-cast onto topographically patterned and planar substrates. A polymer grafting temperature of 230 °C was selected based on previous results.^25,26^Figure S1 presents the TGA showing the thermal degradation temperature of P4VP-OH (250 °C). Figure 2 presents the molecular structure of P4VP-OH and the metal precursors.

The heat treatment facilitates polymer chain repetition and diffusion of the polymer −OH moieties to the substrate surface and subsequent condensation reactions with the substrate OH groups to form covalently bonded polymer films. Complete surface coverage of the polymer brush template is critical in delivering conformal deposition of the high-κ dielectric thin films of controlled thickness. Therefore, a broad area of investigation was performed using CA measurements to examine the thin film deposition of the polymer brush over the entire area of the substrate surface. The water CA images recorded from three representative positions of each film was used to calculate the polymer brush coverage on substrates (see Figure S2). The Cassie Baxter eq 1 with the hypothesis of surface energy homogeneity was used to calculate the surface coverage.
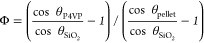
1where Φ is the polymer brush surface coverage, θ_P4VP_ is the advancing water CA of the deposited P4VP brush, θ_SiO_2__ is the advancing water CA observed for the plasma treated SiO_2_ sample, and θ_pellet_ is the advancing water CA of a pure P4VP homopolymer pellet.

The control, oxygen plasma-treated silicon and the silicon surface from the trench patterned samples have an advancing water CA (θ_a_) value of 5 ± 0.5°. The grafted P4VP polymer on flat regions of the patterned substrate and thick spin-coated P4VP films show a similar θ_a_ of 65°. Pelletized P4VP homopolymer θ_a_ values vary from 65 to 73° due to the pressure dependent transition of the P4VP motifs.^40^ The CA images and the data derived by solving the Cassie Baxter equation using the θ_a_ values confirmed a complete P4VP-OH polymer grafting on the substrates (see Figure S2).

Metal uptake was achieved by spin coating ethanolic solutions of metal salts onto the fabricated P4VP-OH monolayer films. The synergistic action of solvent swelling and nitrogen loan pair enhanced binding of the P4VP^41^ with Hf^4+^ and Zr^4+^ created homogeneous distribution of metal cation and counter anion. Subsequently, the continuous metal oxide films were produced by treating the samples under oxygen plasma for 20 min. The plasma treatment enabled the rapid oxidative removal of the polymer brush and conversion of the metal precursor into the respective metal oxide.

**Figure 3:**
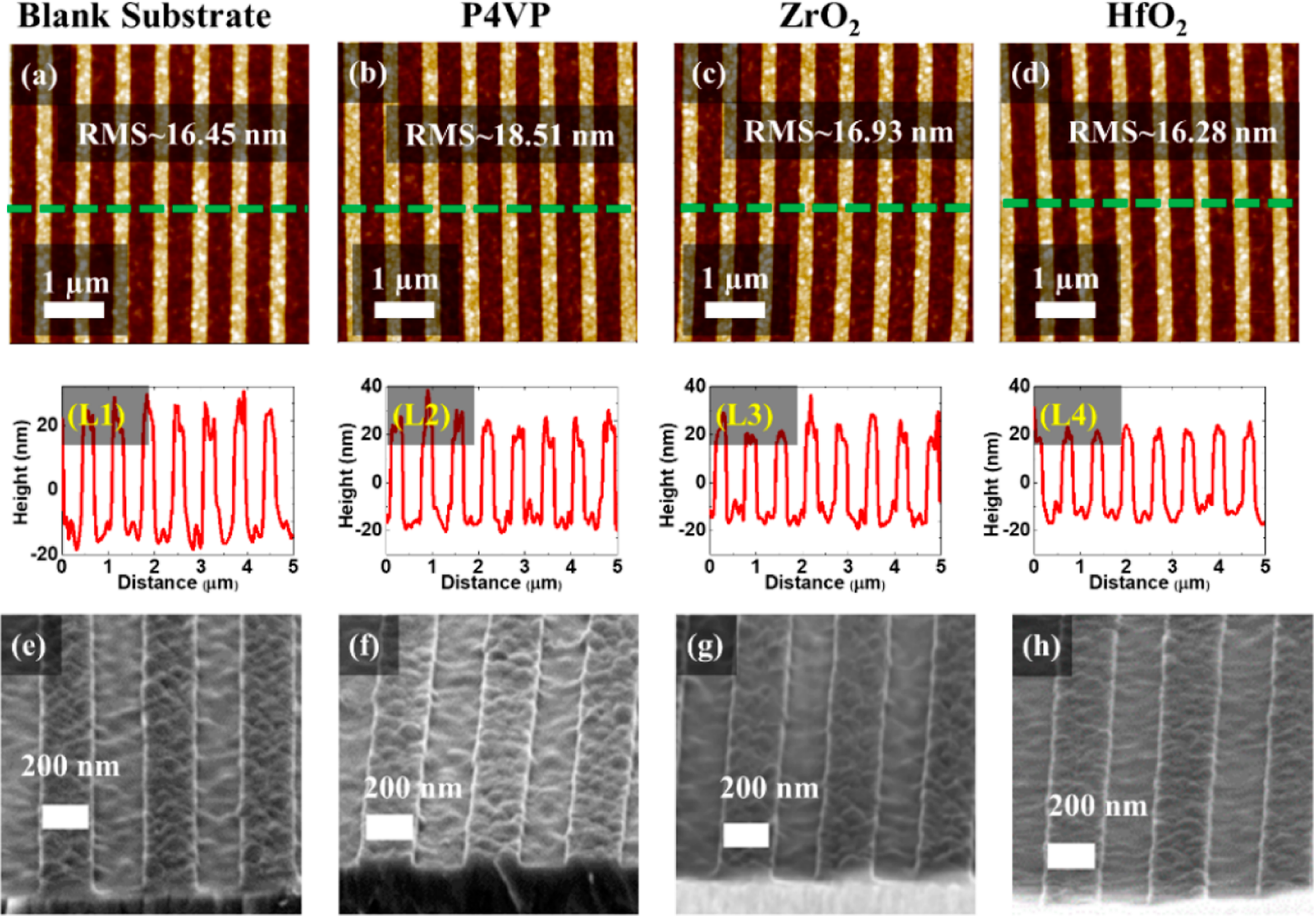
AFM images (a–d) with line profile plotted (L1–L4) and angled SEM images (e–h) for blank substrate (a,L1,e), P4VP grafted brush (b,L2,f), ZrO_2_ (c,L3,g), and HfO_2_ (d,L4,h) films.

AFM was used to examine and compare the roughness and homogeneity of the control uncoated patterned substrates, the grafted P4VP-OH films, and the final ZrO_2_ and HfO_2_ films. All four stages provided similar root mean square (RMS) values (L1 to L4 in Figure 3). The uncoated patterned silicon substrate provided RMS value of 16.45 nm. The P4VP grafted brush had a slightly higher RMS value of 18.51 nm, which is consistent with the polymer brush grafting. The metal oxide films showed a reduced RMS (16.93 nm for ZrO_2_ nm and 16.28 nm for HfO_2_) roughness, similar to that of the uncoated substrate, indicating polymer brush ashing and conformal coating of the metal oxide with uniform thickness. We have compared the roughness values to that of the metal oxide films prepared on native oxide silicon substrates using the P4VP-OH template-assisted depositions. We observed ultra-smooth, uniform films with RMS roughness values in the range of 0.4–0.8 nm (see Figure S3).

The high-resolution angled SEM images presented in Figure 3e,f represent the as-received topographically patterned silicon nitride substrate and conformally grafted P4VP films. Zirconium oxide and hafnium oxide films were developed by infiltrating the ethanolic solution of the precursor (0.4 wt %). The interaction between ionic ethanol and P4VP segments is favorable for the optimum reaction.^42^Figure 3g,h exhibits SEM image of the P4VP-assisted continuous zirconium oxide and hafnium oxide films across the trenches. No obvious defects or roughness is observed for the deposited films. The SEM images recorded from native oxide substrates, presented in Figure S3, also shows the formation of highly uniform films. We expect similar grafting densities for both silicon native oxide substrates and silicon nitride as the roughness values in flat regions of both substrates are similar.

**Figure 4:**
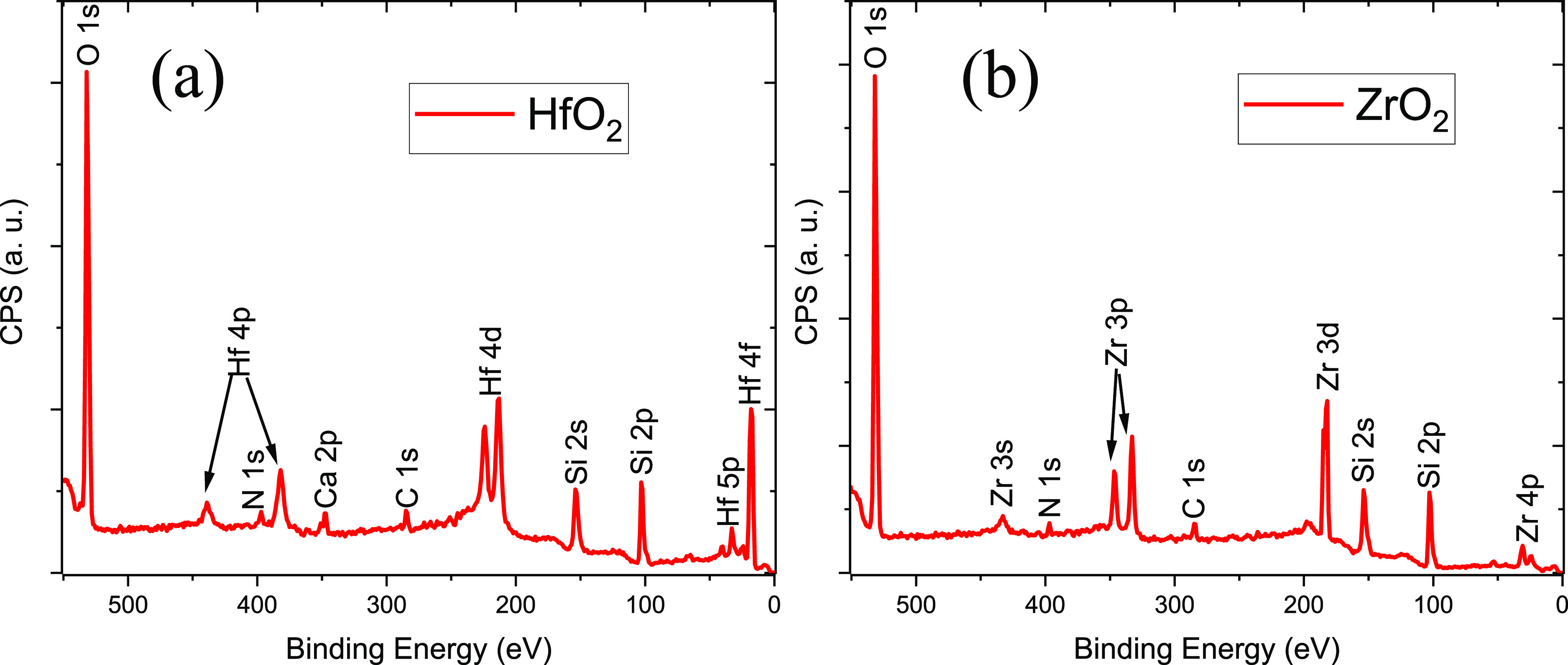
XPS survey spectra for (a) HfO_2_ and (b) ZrO_2_.

XPS survey spectra of the ZrO_2_, Figure 4a, and HfO_2_, Figure 4b, films clearly show that the respective metal is present on the surface. The HfO_2_ sample has peaks that can be assigned to Si (34.16%), O (47.79%), C (6.45%), N (2.47%), and Hf (7.46%). A small amount of Ca is present (1.67%), most likely due to contamination. The ZrO_2_ sample has peaks from Si (33.75%), O (50.52%), C (5.36%), N (1.62%), and Zr (8.75%). In both cases, the C contribution was <5%, which shows the benefit of being able to measure the XPS directly after plasma processing, without exposing the samples to atmospheric conditions.

**Figure 5:**
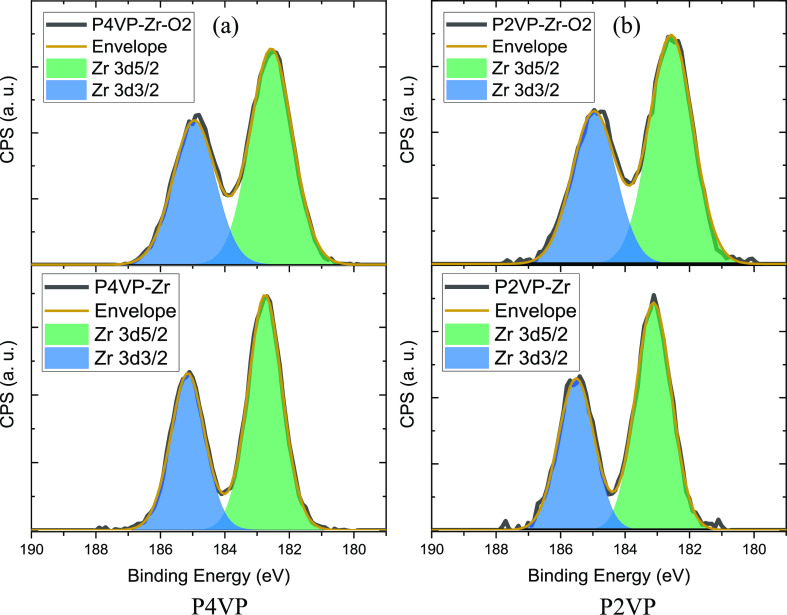
High-resolution XPS spectra of the Zr 3d region pre (bottom) and post (top) O_2_ plasma treatment for P4VP (a) and P2VP (b).

The effect of polymer was investigated by inspection of the high-resolution scans of the Zr 3d region before and after O_2_ plasma processing. Using either P4VP (Figure 5a) or P2VP (Figure 5b) shows that the position of the nitrogen does not affect the chemical environment of the Zr. Two peaks for the ZrN_2_O_7_ infiltrated samples for both P2VP and P4VP located at 183.1 and 185.5 eV are attributed to the Zr 3d_5/2_ and Zr 3d_3/2_ spin–orbit peaks, respectively, as seen in bottom graph Figure 5a. Similar peak positions are observed for Figure 5b with peak separation of 2.4 eV. After the O_2_ plasma exposure, a negative shift of 0.5 eV is observed owing to the formation of ZrO_2_.^43^ The only measurable difference is the amount of metal uptake (higher in P4VP), consistent with steric hindrance of side chain in P2VP and may be a limiting factor.

**Figure 6:**
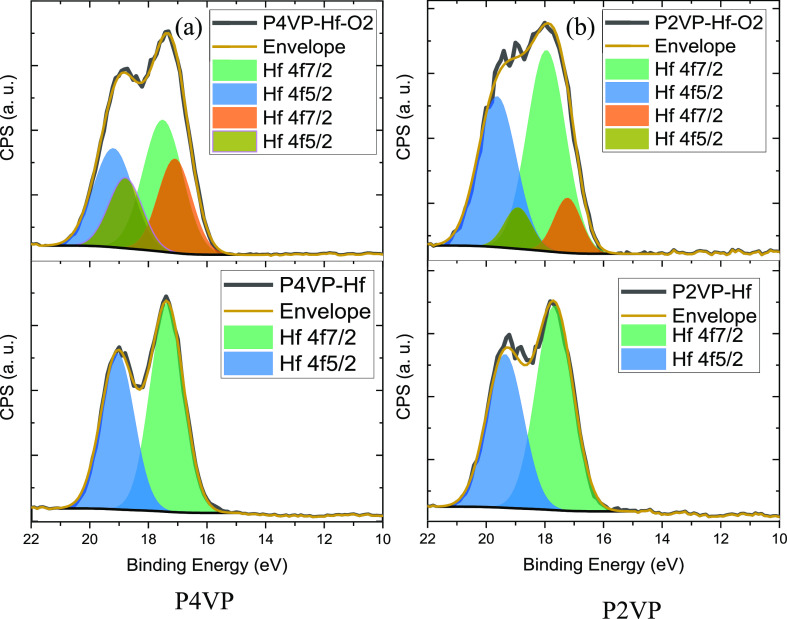
High-resolution XPS spectra of the Hf 4f region pre (bottom) and post (top) O_2_ plasma treatment for P4VP (a) and P2VP (b).

The Hf 5f high-resolution scans are well fitted with a doublet showing HfO_2_ before being exposed to O_2_ plasma (Figure 6, lower plots). After plasma treatment, two distinct chemical environments are revealed—hafnium oxide (HfO_2_) and sub-oxide (HfO_*x*_, *x* < 2).^44^ As seen in the top plots in Figure 6, the HfO_2_ peak is the majority component at 17.5 eV (Hf 4f_7/2_).^45,46^ A second peak is evident at 0.5 eV lower binding energy attributed to sub-oxide species. The spin–orbit splitting between Hf 4f_7/2_ and Hf 4f_5/2_ is approximately 1.69 eV.

**Figure 7:**
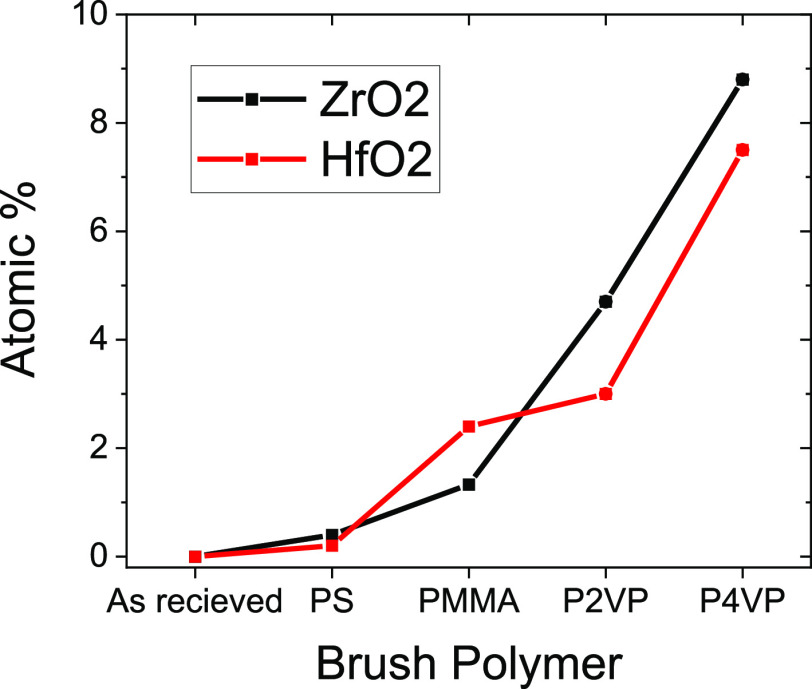
XPS plot of atomic percentage of metal for various polymer systems.

The atomic percentage of Zr and Hf were extracted from XPS survey spectra of the thin films formed when using different polymer brushes. If polystyrene or no polymer was used, there was no corresponding metal oxide film present, as expected because polystyrene is a saturated molecule, and there is no chemical interaction between the polymer and the metal salt. PMMA results in 1.3% Zr and 2.4% Hf, this is likely due to the weak interaction between the metal cation and C=Ö site on the PMMA matrix. P2VP shows around 3.5%, limited by steric hindrance. P4VP is the polymer which displays the greatest amount of metal uptake, with around 8% (Figure 7).

**Figure 8:**
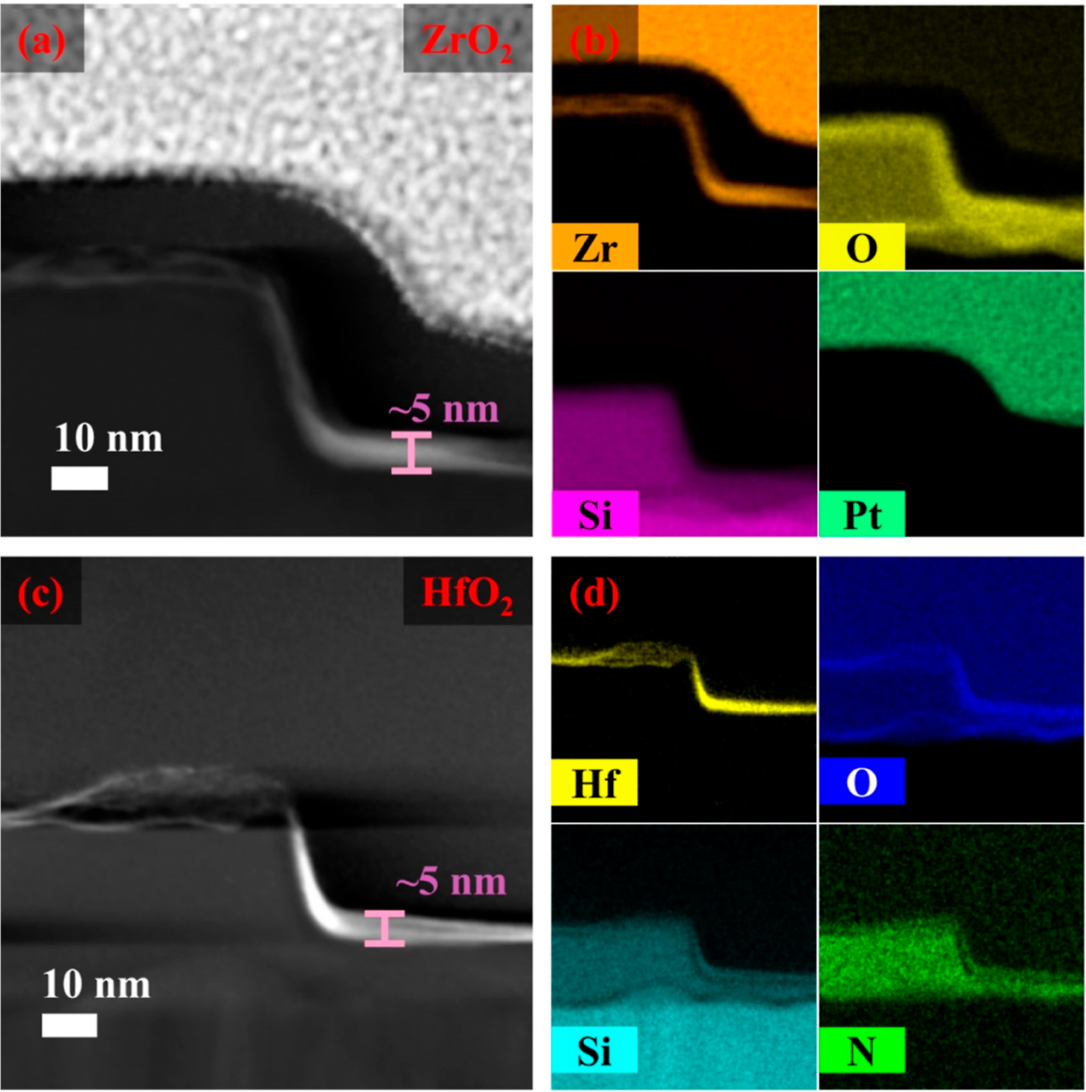
HAADF-STEM images of ZrO_2_- and HfO_2_-coated patterned substrates (a,c) and elemental EDX mapping images of the respective films (b,d).

STEM high-angle annular dark-field imaging (HAADF) was carried out to examine the metal oxide deposition on the trenches and pitches of the substrate following polymer plasma ashing and oxide conversion. The representative HAADF-STEM cross-sectional images (Figure 8a,c) show uniformly deposited ZrO_2_ and HfO_2_ films on a patterned trench. Deposited ZrO_2_ and HfO_2_ films were found to be highly regular across the top, bottom, and sidewall of the trenches. TEM cross section of the coated ZrO_2_ and HfO_2_ (Figure 8a,c) shows the nano metal oxide films of thickness ∼5 nm is formed. Interestingly, HfO_2_ and ZrO_2_ deposition across the trenches establish the capability of the P4VP to grow ordered nano metal oxide films. The EDX mapping images (Figure 8b,d) confirm the presence of Zr, Hf, and O, showing that zirconia and hafnia form uniform thin films over the trenches. The films coat both the flat horizontal and vertical trench areas, indicating that the process allows ubiquitous metal oxide formation throughout. Therefore, our method is advantageous over traditional CVD or ALD as the metal oxides can also coat perpendicularly oriented substrate surfaces to the same degree as flat surfaces. The presence of the nitrogen signal in the EDX mapping is due to native silicon nitride.

The possibility of fabricating thin metal oxide films by varying the molecular weight of the P4VP polymer could be explored. A variety of metal interactions with P4VP are emphasized, including sterically demanding species (e.g., silver and gold), and these metal oxide films can also be produced utilizing our technique.

## Conclusions

A polymer brush template approach that facilitates a controlled inclusion of the metal ions to fabricate uniform sub 5 nm films of metal oxides (HfO_2_ and ZrO_2_) is reported on topographically patterned silicon nitride substrates. The SEM, STEM-HAADF, AFM, and CA data show that high-quality pinhole-free films are uniformly and conformally deposited across the trenches and pitches of patterned substrates. The P4VP-OH brush coating is demonstrated to be optimal for a controlled conformal deposition of ultrathin films on to the trenches and pitches of the substrates, as the polymer in its neutral form coordinates with the metal ions by donating the lone pair electrons of its nitrogen atom with less steric hindrance. Further work is required to understand the feature size limitation of conformal topographic coating. Passivation of the substrate surface against metal inclusion has also been demonstrated using unreactive polystyrene brush showing the potential for selective masking and deposition. It is anticipated that this simple templating technique could allow infiltration of a wide range of materials using liquid and vapor phase techniques on to a wide range of complex topographically patterned substrates. The current technology provided here can facilitate a path toward practical non-planar deposition, with applications to a variety of developing nanostructures.

There are distinct advantages of this solution phase infiltration method versus competitive methods. There are cost and infrastructure benefits based on the simple chemistry. It is extensible to many metal salts as precursors allowing a more varied range of elemental choice based on the strict requisites of, for example, ALD where volatile precursors are needed. Conformal coating is possible even on samples with complex topography because of the “monolayer” nature of the brush deposition which is self-limited to a single layer of polymer molecule. This avoids, for example, accumulation at step features. It can also be used to coat small feature size of 50 nm and less. Limited only to the feature size limited penetration of the solvent. Compared to vacuum techniques, non-line of sight coating can be readily achieved. As well as application in electronic device fabrication, this could be applied to any application requiring thin-film oxide deposition.

## Supporting Information Available

TGA of powder P4VP, CA, AFM, and SEM data (PDF)

## References

[ref1] Stehlin F.; Bourgin Y.; Spangenberg A.; Jourlin Y.; Parriaux O.; Reynaud S.; Wieder F.; Soppera O. Direct Nanopatterning of 100 Nm Metal Oxide Periodic Structures by Deep-UV Immersion Lithography. Opt. Lett. 2012, 37, 4651 10.1364/ol.37.004651. 23164868

[ref2] Englard I.; Stegen R.; Vanoppen P.; Minnaert-Janssen I.; der Kinderen T.; van Brederode E.; Duray F.; Linders J.; Ovchinnikov D.; Piech R.; Masia C.; Hillel N.; Ravid E.; Rotlevi O.; Wilde A.; Shabtay S.; Telor Z.; Schreutelkamp R. Novel Approach for Immersion Lithography Defectivity Control to Increase Productivity. Metrology, Inspection, and Process Control for Microlithography XXII, 2008; Vol. 6922, p 69223U.

[ref3] Hasan R. M. M.; Luo X. Promising Lithography Techniques for Next-Generation Logic Devices. Nanomanuf. Metrol. 2018, 1, 67–81. 10.1007/s41871-018-0016-9.

[ref4] Keyes R. W. Miniaturization of Electronics and Its Limits. IBM J. Res. Dev. 1988, 32, 84–88. 10.1147/rd.321.0024.

[ref5] Ratnesh R. K.; Goel A.; Kaushik G.; Garg H.; Chandan; Singh M.; Prasad B. Advancement and Challenges in MOSFET Scaling. Mater. Sci. Semicond. Process. 2021, 134, 106002 10.1016/j.mssp.2021.106002.

[ref6] Jiang X.; Bent S. F. Area-Selective ALD with Soft Lithographic Methods: Using Self-Assembled Monolayers to Direct Film Deposition. J. Phys. Chem. C 2009, 113, 17613–17625. 10.1021/jp905317n.

[ref7] Minaye Hashemi F. S.; Birchansky B. R.; Bent S. F. Selective Deposition of Dielectrics: Limits and Advantages of Alkanethiol Blocking Agents on Metal-Dielectric Patterns. ACS Appl. Mater. Interfaces 2016, 8, 33264–33272. 10.1021/acsami.6b09960. 27934166

[ref8] Mackus A. J. M.; Merkx M. J. M.; Kessels W. M. M. From the Bottom-Up: Toward Area-Selective Atomic Layer Deposition with High Selectivity. Chem. Mater. 2019, 31, 2–12. 10.1021/acs.chemmater.8b03454. 30774194 PMC6369656

[ref9] Chen R.; Li Y.-C.; Cai J.-M.; Cao K.; Lee H.-B.-R. Atomic level deposition to extend Moore’s law and beyond. Int. J. Extreme Manuf. 2020, 2, 022002 10.1088/2631-7990/ab83e0.

[ref10] Färm E.; Vehkamäki M.; Ritala M.; Leskelä M. Passivation of Copper Surfaces for Selective-Area ALD Using a Thiol Self-Assembled Monolayer. Semicond. Sci. Technol. 2012, 27, 074004 10.1088/0268-1242/27/7/074004.

[ref11] Nagata T. Material Design of Metal/Oxide Interfaces for Nanoelectronics Applications, 1st ed.; Springer Japan, 2020.

[ref12] Prasittichai C.; Pickrahn K. L.; Minaye Hashemi F. S.; Bergsman D. S.; Bent S. F. Improving Area-Selective Molecular Layer Deposition by Selective SAM Removal. ACS Appl. Mater. Interfaces 2014, 6, 17831–17836. 10.1021/am504441e. 25290370

[ref13] Mameli A.; Merkx M. J. M.; Karasulu B.; Roozeboom F.; Kessels W. M. M.; Mackus A. J. M. Area-Selective Atomic Layer Deposition of SiO2 Using Acetylacetone as a Chemoselective Inhibitor in an ABC-Type Cycle. ACS Nano 2017, 11, 9303–9311. 10.1021/acsnano.7b04701. 28850774 PMC5665545

[ref14] Mameli A.; Karasulu B.; Verheijen M. A.; Barcones B.; Macco B.; Mackus A. J. M.; Kessels W. M. M. E.; Roozeboom F. Area-Selective Atomic Layer Deposition of ZnO by Area Activation Using Electron Beam-Induced Deposition. Chem. Mater. 2019, 31, 1250–1257. 10.1021/acs.chemmater.8b03165.

[ref15] Chen R.; Kim H.; McIntyre P. C.; Porter D. W.; Bent S. F. Achieving Area-Selective Atomic Layer Deposition on Patterned Substrates by Selective Surface Modification. Appl. Phys. Lett. 2005, 86, 191910 10.1063/1.1922076.

[ref16] Singh J. A.; Thissen N. F. W.; Kim W.-H.; Johnson H.; Kessels W. M. M.; Bol A. A.; Bent S. F.; Mackus A. J. M. Area-Selective Atomic Layer Deposition of Metal Oxides on Noble Metals through Catalytic Oxygen Activation. Chem. Mater. 2018, 30, 663–670. 10.1021/acs.chemmater.7b03818. 29503508 PMC5828705

[ref17] Färm E.; Kemell M.; Ritala M.; Leskelä M. Self-Assembled Octadecyltrimethoxysilane Monolayers Enabling Selective-Area Atomic Layer Deposition of Iridium. Chem. Vap. Deposition 2006, 12, 415–417. 10.1002/cvde.200604219.

[ref18] Chen R.; Kim H.; McIntyre P. C.; Bent S. F. Self-assembled monolayer resist for atomic layer deposition of HfO2 and ZrO2 high-κ gate dielectrics. Appl. Phys. Lett. 2004, 84, 4017–4019. 10.1063/1.1751211.

[ref19] Zhao Y.; Sun X. Molecular Layer Deposition for Energy Conversion and Storage. ACS Energy Lett. 2018, 3, 899–914. 10.1021/acsenergylett.8b00145.

[ref20] Kim W.-H.; Minaye Hashemi F. S.; Mackus A. J. M.; Singh J.; Kim Y.; Bobb-Semple D.; Fan Y.; Kaufman-Osborn T.; Godet L.; Bent S. F. A Process for Topographically Selective Deposition on 3D Nanostructures by Ion Implantation. ACS Nano 2016, 10, 4451–4458. 10.1021/acsnano.6b00094. 26950397

[ref21] Stevens E.; Tomczak Y.; Chan B. T.; Altamirano Sanchez E.; Parsons G. N.; Delabie A. Area-Selective Atomic Layer Deposition of TiN, TiO _2_ , and HfO _2_ on Silicon Nitride with Inhibition on Amorphous Carbon. Chem. Mater. 2018, 30, 3223–3232. 10.1021/acs.chemmater.8b00017.

[ref22] Atanasov S. E.; Kalanyan B.; Parsons G. N. Inherent Substrate-Dependent Growth Initiation and Selective-Area Atomic Layer Deposition of TiO _2_ Using “Water-Free” Metal-Halide/Metal Alkoxide Reactants. J. Vac. Sci. Technol., A 2016, 34, 01A148 10.1116/1.4938481.

[ref23] Cummins C.; Weingärtner T.; Morris M. A. Enabling Large-Area Selective Deposition on Metal-Dielectric Patterns Using Polymer Brush Deactivation. J. Phys. Chem. C 2018, 122, 14698–14705. 10.1021/acs.jpcc.8b04092.

[ref24] Cummins C.; Shaw M. T.; Morris M. A. Area Selective Polymer Brush Deposition. Macromol. Rapid Commun. 2017, 38, 1700252 10.1002/marc.201700252. 28671756

[ref25] Lundy R.; Yadav P.; Selkirk A.; Mullen E.; Ghoshal T.; Cummins C.; Morris M. A. Optimizing Polymer Brush Coverage To Develop Highly Coherent Sub-5 Nm Oxide Films by Ion Inclusion. Chem. Mater. 2019, 31, 9338–9345. 10.1021/acs.chemmater.9b02856.

[ref26] Lundy R.; Yadav P.; Prochukhan N.; Giraud E. C.; O’Mahony T. F.; Selkirk A.; Mullen E.; Conway J.; Turner M.; Daniels S.; Mani-Gonzalez P. G.; Snelgrove M.; Bogan J.; McFeely C.; O’Connor R.; McGlynn E.; Hughes G.; Cummins C.; Morris M. A. Precise Definition of a “Monolayer Point” in Polymer Brush Films for Fabricating Highly Coherent TiO2Thin Films by Vapor-Phase Infiltration. Langmuir 2020, 36, 12394–12402. 10.1021/acs.langmuir.0c02512. 33021792

[ref27] Kang Y. H.; Lee S.; Choi Y.; Seong W. K.; Han K. H.; Kim J. H.; Kim H. M.; Hong S.; Lee S. H.; Ruoff R. S.; Kim K. B.; Kim S. O. Large-Area Uniform 1-Nm-Level Amorphous Carbon Layers from 3D Conformal Polymer Brushes. A “Next-Generation” Cu Diffusion Barrier?. Adv. Mater. 2022, 34, 2110454 10.1002/adma.202110454. 35085406

[ref28] Goldberger J.; Hochbaum A. I.; Fan R.; Yang P. Silicon Vertically Integrated Nanowire Field Effect Transistors. Nano Lett. 2006, 6, 973–977. 10.1021/nl060166j.

[ref29] Kim J.; Park J.; Pham D. P.; Yeo M. S.; Rhee H.; Kim Y.-S.; Cho E.-C.; Yi J. Combination of Ultraviolet Exposure and Thermal Post-Treatment to Obtain High Quality HfO2 Thin Films. Ceram. Int. 2021, 47, 9643–9650. 10.1016/j.ceramint.2020.12.103.

[ref30] Jindal S.; Manhas S. K.; Gautam S. K.; Balatti S.; Kumar A.; Pakala M. Investigation of Gate-Length Scaling of Ferroelectric FET. IEEE Trans. Electron Devices 2021, 68, 1364–1368. 10.1109/TED.2021.3054720.

[ref31] Kennemur J. G. Poly(Vinylpyridine) Segments in Block Copolymers: Synthesis, Self-Assembly, and Versatility. Macromolecules 2019, 52, 1354–1370. 10.1021/acs.macromol.8b01661.

[ref32] Mokarian-Tabari P.; Senthamaraikannan R.; Glynn C.; Collins T. W.; Cummins C.; Nugent D.; O’Dwyer C.; Morris M. A. Large Block Copolymer Self-Assembly for Fabrication of Subwavelength Nanostructures for Applications in Optics. Nano Lett. 2017, 17, 2973–2978. 10.1021/acs.nanolett.7b00226. 28379701

[ref33] Peng Q.; Tseng Y.-C.; Darling S. B.; Elam J. W. A Route to Nanoscopic Materials via Sequential Infiltration Synthesis on Block Copolymer Templates. ACS Nano 2011, 5, 4600–4606. 10.1021/nn2003234. 21545142

[ref34] Zhang Z.; Dwyer T.; Sirard S. M.; Ekerdt J. G. Area-Selective Atomic Layer Deposition of Cobalt Oxide to Generate Patterned Cobalt Films. J. Vac. Sci. Technol., A 2019, 37, 020905 10.1116/1.5066437.

[ref35] IEEE. IEEE International Interconnect Technology Conference/Advanced Metallization Conference (IITC/AMC), 2016; pp 133–135.

[ref36] Lindvig T.; Michelsen M. L.; Kontogeorgis G. M. A Flory-Huggins Model Based on the Hansen Solubility Parameters. Fluid Phase Equilib. 2002, 203, 247–260. 10.1016/S0378-3812(02)00184-X.

[ref37] Hansen C. M. Hansen Solubility Parameters: A User’s Handbook; CRC Press, 2007.

[ref38] Barr T. L.; Seal S. Nature of the Use of Adventitious Carbon as a Binding Energy Standard. J. Vac. Sci. Technol., A 1995, 13, 1239–1246. 10.1116/1.579868.

[ref39] Zheng T.; Zhu M.; Waqas M.; Umair A.; Zaheer M.; Yang J.; Duan X.; Li L. P4VP-RuII(Bda) Polyelectrolyte-Metal Complex as Water Oxidation Catalyst: On the Unique Slow-Diffusion and Multi-Charge Effects of the Polyelectrolyte Ligand. RSC Adv. 2018, 8, 38818–38830. 10.1039/c8ra08012g. 35558290 PMC9090605

[ref40] Raczkowska J.; Stetsyshyn Y.; Awsiuk K.; Zemła J.; Kostruba A.; Harhay K.; Marzec M.; Bernasik A.; Lishchynskyi O.; Ohar H.; Budkowski A. Temperature-Responsive Properties of Poly(4-Vinylpyridine) Coatings: Influence of Temperature on the Wettability, Morphology, and Protein Adsorption. RSC Adv. 2016, 6, 87469–87477. 10.1039/c6ra07223b.

[ref41] Hiruma Y.; Yoshikawa K.; Haga M.-a. Bio-Inspired Protonic Memristor Devices Based on Metal Complexes with Proton-Coupled Electron Transfer. Faraday Discuss. 2019, 213, 99–113. 10.1039/c8fd00098k. 30375604

[ref42] Kumar L.; Horechyy A.; Bittrich E.; Nandan B.; Uhlmann P.; Fery A. Amphiphilic Block Copolymer Micelles in Selective Solvents: The Effect of Solvent Selectivity on Micelle Formation. Polymers 2019, 11, 1882 10.3390/polym11111882. PMC6918162 31739558

[ref43] Wang X.; Xu J.; Quan X.; Li Y.; Wang Y.; Cheng X. Fast Fabrication of Silicon Nanopillar Array Using Electron Beam Lithography with Two-Layer Exposure Method. Microelectron. Eng. 2020, 227, 111311 10.1016/j.mee.2020.111311.

[ref44] Tien T.-C.; Lin L.-C.; Lee L.-S.; Hwang C.-J.; Maikap S.; Shulga Y. M. Analysis of weakly bonded oxygen in HfO2/SiO2/Si stacks by using HRBS and ARXPS. J. Mater. Sci.: Mater. Electron. 2010, 21, 475–480. 10.1007/s10854-009-9941-0.

[ref45] Ohtsu N.; Tsuchiya B.; Oku M.; Shikama T.; Wagatsuma K. X-Ray Photoelectron Spectroscopic Study on Initial Oxidation of Hafnium Hydride Fractured in an Ultra-High Vacuum. Appl. Surf. Sci. 2007, 253, 6844–6847. 10.1016/j.apsusc.2007.01.117.

[ref46] Morant C.; Galán L.; Sanz J. M. An XPS Study of the Initial Stages of Oxidation of Hafnium. Surf. Interface Anal. 1990, 16, 304–308. 10.1002/sia.740160163.

