# Supplementary material for: Fabrication of High-κ Dielectric Metal Oxide Films on Topographically Patterned Substrates: Polymer Brush-Mediated Depositions
Source: ACS Appl Mater Interfaces. 2022 Jul 7;14(28):32729–37. doi: 10.1021/acsami.2c07966 (PMC9305981; doi:10.1021/acsami.2c07966)
Supplement: Supplementary file 1 — am2c07966_si_001.pdf [file am2c07966_si_001.pdf]

Supporting Information

Fabrication of High-κ Dielectric Metal Oxide Films on Topographically Patterned Substrates: Polymer Brush Mediated Depositions

Pravind Yadav, *[1] Riley Gatensby,[1] Nadezda Prochukhan,[1] Sibu C. Padmanabhan,[1] Arantxa Davó-Quiñonero,[1] Philip Darragh,[1] Ramsankar Senthamaraikannan,[1] Bríd Murphy,[1] Matthew Snelgrove,[2] Caitlin McFeely,[2] Sajan Singh,[1] Jim Conway,[3] Robert O'Connor,[2] Enda McGlynn,[2,3] Ross Lundy,[1] and Michael A. Morris*[1]

[1]AMBER Research Centre and School of Chemistry, Trinity College Dublin, Dublin, Ireland

[2]School of Physical Sciences, Dublin City University, Glasnevin, Dublin 9, Ireland

[3]National Centre for Plasma Science and Technology, Dublin City University, Glasnevin, Dublin 9, Ireland

*** Corresponding author: * E-mail:** pyadav@tcd.ie **(P.Y.)**
morrism2@tcd.ie **(M.A.M.)**

The upper threshold temperature for polymer brush grafting was determined using thermogravimetric analysis of powder P4VP-OH. We set a grafting temperature of 230 ^{0}C, to achieve complete monolayer coverage of the polymer brush.

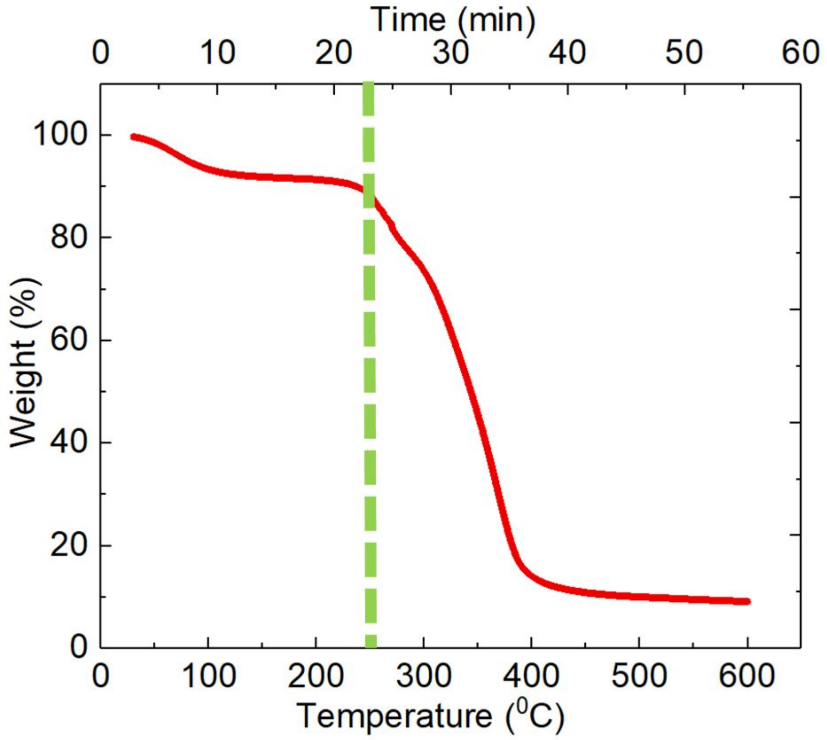

Figure S1 Thermogravimetric analysis was performed on the 5.5 kmol^{-1} P4VP-OH at temperature range of 25-600^{0}C for 60 minute. Polymer undergo thermal degradation at 250^{0}C indicating optimum temperature for the grafting process.

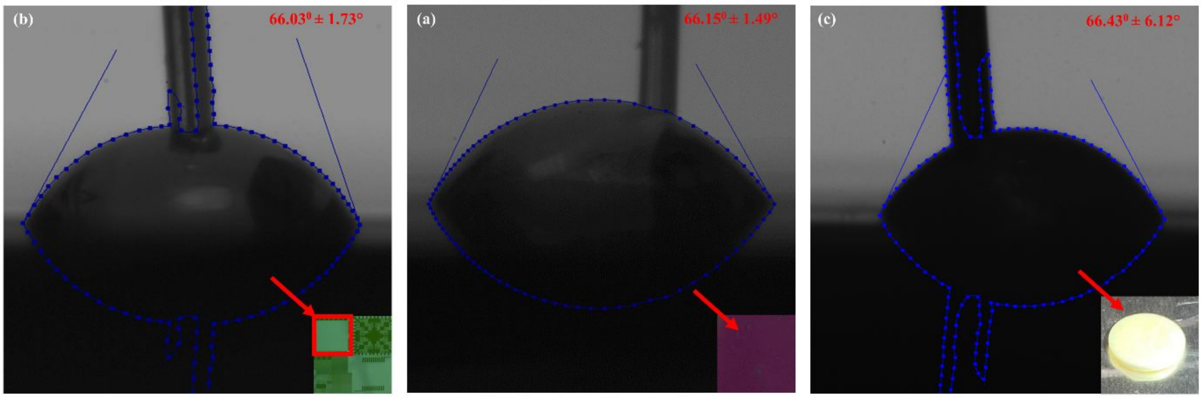

Figure S2 (a) water contact angle angle of grafted P4VP polymer brush measured at plane region of topographically patterned substrate of thick-coated P4VP film on plane silicon substrate **(b)** water contact angle of thick-coated P4VP film on plane silicon substrate **(c)** water contact angle of P4VP homopolymer pressed pellet using hydraulic pressure.

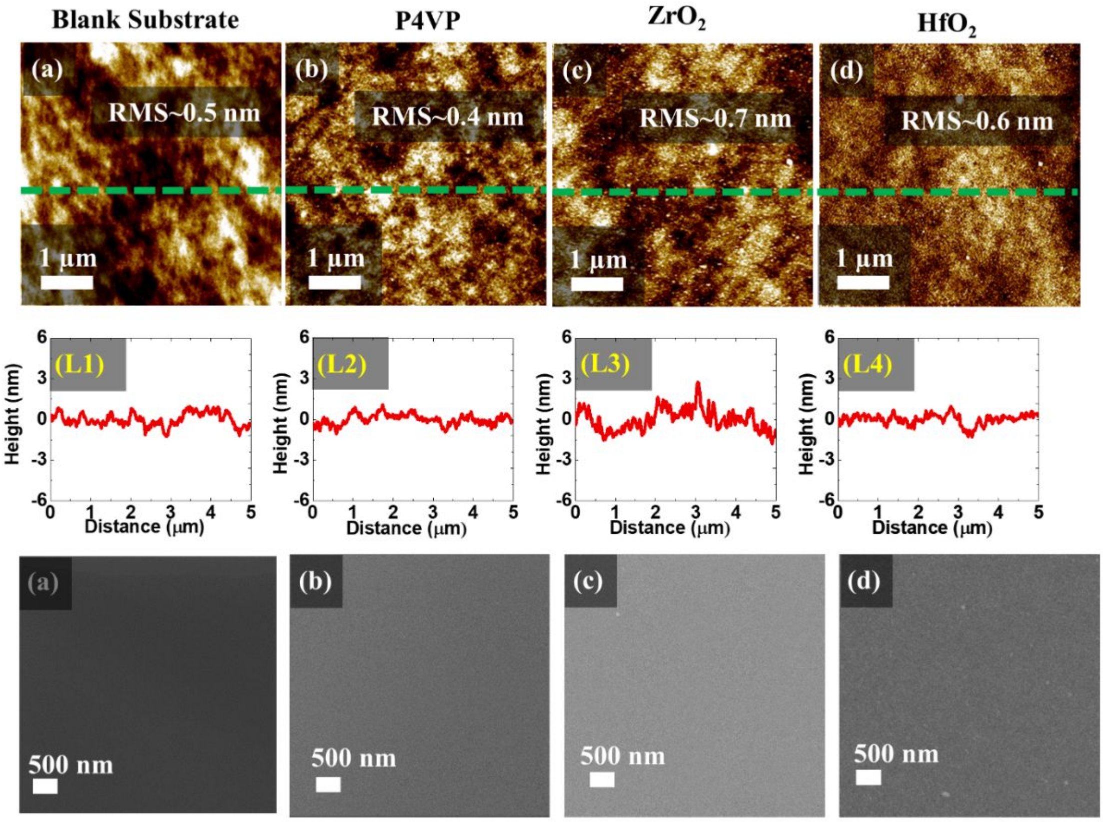

Figure S3 AFM image of **(a)** as receive silicon native oxide substrate with line images at L1. **(b)** AFM and SEM images of P4VP polymer grafted silicon substrates with line images at L2 **(c and d)** are AFM and SEM images of oxidised ZrO_2 and HfO_2 samples with line images at L3 and L4
